# Examining coverage, content, and impact of maternal nutrition interventions: the case for quality-adjusted coverage measurement

**DOI:** 10.7189/jogh.10.010501

**Published:** 2020-06

**Authors:** Naima T Joseph, Ellen Piwoz, Dennis Lee, Address Malata, Hannah H Leslie, on behalf of the Countdown Coverage Technical Working Group

**Affiliations:** 1 Division of Maternal Fetal Medicine, Department of Gynecology and Obstetrics, Emory University School of Medicine, Atlanta, Georgia, USA; 2 Bill and Melinda Gates Foundation, Seattle, Washington, USA; 3 Department of Health Policy and Management, Harvard TH Chan School of Public Health, Boston, Massachusetts, USA; 4 Malawi University of Science and Technology, Limbe, Malawi; 5 Department of Global Health and Population, Harvard TH Chan School of Public Health, Boston, Massachusetts, USA

## Abstract

**Background:**

Reductions in neonatal mortality remain stagnant, despite gains in health care access and utilization. Nutrition interventions during antenatal care (ANC) and in the immediate postpartum period are associated with improved neonatal outcomes. Adjusting coverage estimates for the quality of care provided yields greater insight into health system performance and potential population health benefits of accessing care. In this cross-sectional study, we adjust maternity care coverage measures for quality of nutrition interventions to determine the impact on infant birth weight and breastfeeding.

**Methods:**

We used household data from the Malawi 2013-2014 Multiple Indicator Cluster Survey to assess use of maternal health services and direct observations of ANC and delivery from the 2013 Service Provision Assessment to measure nutrition interventions provided. We adjusted coverage measures combining self-reported utilization of care with the likelihood of receipt of nutrition interventions. Using adjusted log-linear regression, we estimated the associations of these nutrition quality-adjusted metrics with infant birthweight and immediate breastfeeding.

**Results:**

Health facility data provided over 2500 directly observed clinical encounters and household data provided 7385 individual reports of health care utilization and outcomes. Utilization of ANC and facility-delivery was high. Women received nutrition-related interventions considerably less often than they sought care: over the course of ANC women received a median of 1.6 interventions on iron, 1 instance of nutrition counseling, and 0.06 instances of breastfeeding counseling. Nutrition quality-adjusted ANC coverage was associated with a reduced risk of low birthweight (adjusted relative risk [ARR] 0.87, 95% confidence interval (CI) = 0.79, 0.96) and increased likelihood of immediate breastfeeding (ARR = 1.04, 95% CI = 1.02, 1.07); nutrition quality-adjusted post-delivery care was also associated with greater uptake of immediate breastfeeding (ARR = 1.08, 95% CI = 1.02, 1.14). Based on these models, delivering nutrition interventions consistently within the existing level of coverage would decrease population prevalence of low birthweight from 13.7% to 10.8% and increase population prevalence of immediate breastfeeding from 75.9% to 86.0%.

**Conclusions:**

Linking household survey data to health service provision assessments demonstrates that despite high utilization of maternal health services in Malawi, low provision of nutrition interventions is undermining infant health. Substantial gains in newborn health are possible in Malawi if quality of existing services is strengthened.

Despite the dramatic declines in overall child mortality in low- and middle-income countries, progress towards reduction in neonatal mortality remains slow. With an estimated 2.7 million annual deaths, neonatal mortality now comprises 60% of deaths in the first year of life and 40% of deaths in children under 5 [1,2].

Maternal and childhood undernutrition is thought to underlie 45% of neonatal mortality [3-5]. Interventions to improve maternal and infant nutrition such as dietary counseling, iron supplementation, and immediate/exclusive breastfeeding, have been associated with improved pregnancy and neonatal outcomes [1,6-10]. It is estimated that these interventions could avert more than 800 000 deaths in children under age 5 each year [11]. Iron supplementation has been demonstrated to reduce low birth weight, and immediate breastfeeding is associated with reduced neonatal mortality [1,12]. In addition to neonatal outcomes, breastfeeding has been associated with multiple child health benefits, including reduced overall child mortality, as well as reduction in childhood obesity, asthma, diabetes, and infection [13,14]. Maternal benefits include reductions in type II diabetes and in breast and ovarian cancer [13]. Failure to counsel women on the proximal and long-lasting maternal, neonatal, and child health benefits of iron supplementation and breastfeeding during antenatal care represents missed opportunity for improving long-term maternal and child health. Yet, antenatal coverage and counseling to support these practices are not consistently delivered or measured [1,12].

In 2016, the World Health Organization (WHO) reviewed their antenatal care (ANC) guidelines and reaffirmed their recommendations for dietary counseling, maternal iron supplementation, and exclusive breastfeeding within the context of routine ANC [15]. Additionally, WHO updated their guidelines to increase the frequency of ANC contacts from four to eight, emphasizing the importance of ANC in improving neonatal health outcomes [15]. Utilization of ANC services is associated with reduced probability of adverse neonatal outcomes such as mortality, low birth weight, and stunting [16]. The prioritization of increased access to health facilities and health care coverage during the Millennium Development Goal era led to dramatic increase in ANC utilization [17,18]. By 2015, 90% of women in the countries with 95% of global maternal and child mortality attended at least one ANC visit [17]; over half of women attended at least 4 ANC visits, and 3 out of 4 women delivered with a skilled birth attendant [19]. However, decreases in neonatal mortality did not keep pace with increased utilization of care; annual declines in neonatal mortality will need to double in high-mortality countries to achieve ambitious global targets by 2030 [19].

Analyses of the Demographic and Health Surveys (DHS) and self-reported evaluation on ANC content revealed missed opportunities to improve nutrition and breastfeeding in pregnant women. The focus on improving access to ANC, without ensuring adequate content of care, is insufficient to reducing adverse neonatal outcomes [9,20-24]. A recent analysis of health care-amenable mortality estimated that the majority (657 555 of 1 080 817, 60.8%) of amenable deaths in newborns were attributable to poor quality care rather than lack of access to care [25]. The current effort to achieve the Sustainable Development Goals will require more robust attention to the quality of care provided, as well as to improvements in how these services are measured [18,26].

Current global measurement focuses on crude coverage indicators such as whether or not a woman received any antenatal care or whether or not a skilled birth attendant was present at delivery, measures which are self-reported through household surveys [19]. Effective coverage is a metric that unites need, use, and quality to more reliably capture the relationship between health service delivery and population health outcomes [27,28]. Current literature uses a range of quality measures – including measures of structure [29,30], process of care [31,32], and health outcomes [33] – to calculate effective coverage or quality-adjusted coverage. The specific focus of our analysis is adjusting crude maternal care coverage for measures of nutrition intervention content and quality. Adjusting coverage estimates for the quality of care provided can yield greater insight into health system performance and potential population health benefits of accessing care. However, the data on valid quality measures are sparse and more challenging to obtain than self-reported health care utilization measures: household surveys address content of care inconsistently [34], and maternal recall of specific interventions is highly variable by intervention, context, and survey timing [35-37]. Given the known benefit of maternal nutrition intervention on neonatal outcomes such as birthweight and breastfeeding, we use health system and population information to define nutrition quality-adjusted coverage metrics and quantify their impact on breastfeeding and birthweight.

## METHODS

We used household survey data to generate estimates of health care utilization among those in need (crude coverage), and health facility clinical observation data to summarize the content of care provided in the service environment accessible to each woman. We linked crude coverage and service environment information to adjust individual-level coverage metrics for quality of nutrition interventions. Malawi was used as the case study for testing this approach due to the country’s success in achieving the MDG child mortality target, the high utilization of maternal health care, and the availability of both population and health facility data [38].

### Data sources and study sample

Data on women with recent births or young children were obtained from the 2013-2014 Malawi Multiple Indicator Cluster Survey (MICS), a nationally representative survey conducted in collaboration between the Malawi government and the United Nations Children’s Fund (UNICEF). Households were selected from previously defined enumeration areas (EAs) based on the 2008 census. Of 25 430 eligible women aged 15-49, 24 230 were successfully interviewed (95.3% response rate). We extracted information on household, maternal, and child characteristics for live births in the preceding two years. We obtained the exact spatial location of EA centroids from the Malawi National Statistical Office in order to link the EA location with health facilities providing antenatal and delivery care.

Data on health facilities and services were obtained from the 2013 Malawi Service Provision Assessment (SPA), a census of formal-sector health facilities conducted by the Demographic and Health Surveys (DHS) program. Geospatial coordinates were collected for all facilities, as were urban vs rural location, district, and region name. We divided facilities based on district and urban location to match the 31 districts and cities included in the MICS.

For facilities providing ANC, with or without delivery care, the SPA survey team conducted direct observation of ANC visits and interviews with women following ANC. Visits were sampled for inclusion using systematic random sampling based on the number of clients present during the day of the assessment, with a goal of sampling a maximum of five visits per provider and fifteen per facility. Interviewers attempted to oversample first ANC visits if possible; for this analysis, ANC visits were defined as first, second, third, fourth or later based on client report. The survey team attempted to complete a direct observation of normal delivery at facilities providing normal delivery care. Observations for either ANC or delivery could not be conducted if services were available on the day of the assessment, but no clients presented for care.

### Infant and child outcomes

We defined two outcomes that should be affected by nutritional interventions during ANC for which data are available from the MICS questionnaire: birthweight and immediate breastfeeding.

Infant birthweight in kilograms was defined from maternal recall; the survey team verified weight from infant health cards where available. In keeping with prior analyses, we used multiple imputation to estimate birthweight for missing values [39]; we used a linear regression model using covariates of child size (categorical, based on maternal recall), neonatal death and male sex as well as the exposures and covariates of the analytic model defined below and generated 5 imputed data sets. Low birthweight was defined as birthweight less than or equal to 2.5 kg. Birthweight analysis was restricted to singleton births; each delivery resulting in multiple children was included as a single observation for the analysis of immediate breastfeeding.

Immediate breastfeeding was defined based on maternal report of putting the infant to breast within 1 hour of birth.

### Crude coverage

Crude ANC coverage was defined based on women’s self-reported number of ANC visits in the MICS. Intrapartum care coverage was defined as self-report of childbirth at a formal health facility.

### Quality of nutrition interventions at health facilities

We used direct observation of ANC and delivery care from the SPA to estimate how frequently facilities provided evidence-based nutrition interventions.

We identified three nutrition interventions that should be delivered during ANC: provision of iron-folic acid (IFA) supplements and counseling on their side effects, counseling on appropriate nutrition and diets during pregnancy, and counseling and support for early and exclusive breastfeeding (the direct observation protocol in the SPA does not provide a specific time period such as within 1 hour for the content of counseling on early breastfeeding). Each ANC observation was scored from 0 to 1 on each of these three interventions; we calculated provision of each intervention at the facility level by averaging scores across observations with the same visit number (first visit, second visit, etc.). We also calculated average adherence at the district level and imputed missing facility values with the district average when a facility had no visits of a particular number or no visits at all observed. We added up expected interventions across visit numbers for each facility to calculate the number of times a woman seeking ANC for a given number of visits at that facility could expect to receive each evidence-based nutrition intervention.

Similarly, we identified post-delivery interventions to improve breastfeeding uptake. The primary measure of interest was direct observation of breastfeeding initiation within 1 hour of delivery. We defined a secondary measure based on observation of breastfeeding initiation, the newborn being placed skin-to-skin if breathing, and keeping the mother and newborn in the same room. Each delivery observation was scored as 0 or 1 for immediate breastfeeding and then from 0 to 1 based on performance of these three actions. Facility scores were calculated as the average score across observations.

### Calculation of adjusted coverage: Antenatal care

The MICS does not provide information on the type of health facility where women sought ANC or other details that would enable matching women to a specific facility. We therefore calculated intervention provision based on the health service environment for each EA. The national health policy of Malawi defines 8 km as the maximum acceptable distance to a facility providing the essential health package (including reproductive health care); we therefore defined the health service environment for ANC as the health facilities providing ANC services within 8 km of the patient’s EA, or the single nearest ANC facility if none are within that distance. In urban areas with many health facilities, we limited the service environment to 5 km due to the large number of facilities available to women.

We averaged expected intervention provision scores per number of ANC visits across the service environment, weighting facilities by the ANC patient volume on the day of the visit to reflect greater use of larger facilities, like hospitals, than less busy clinics. We averaged service environment quality scores for IFA and ANC nutrition counseling to capture health system contributions to birthweight for a given number of ANC visits, and we averaged cumulative scores for nutrition counseling and breastfeeding support as inputs contributing to breastfeeding outcomes. These scores capture the expected provision of nutrition interventions for a given number of ANC visits in a specific geographic area.

Each woman in the MICS with a live birth in the past 2 years was assigned the adjusted coverage score corresponding to the number of ANC visits she reported. Quality-adjusted coverage scores could range from 0 (no ANC or no interventions delivered) to the number of ANC visits received (all interventions delivered at all visits), with women reporting four or more visits assigned the same score.

### Calculation of adjusted coverage: Post-delivery care

Childbirth services are offered at 540 health facilities in Malawi, but at 318 facilities no women gave birth on the day of the SPA visit, leaving 222 facilities with at least one directly observed delivery for analysis. We therefore calculated quality-adjusted coverage of post-delivery nutrition interventions at the district (as opposed to EA) level. We averaged the facility scores for these interventions by district, stratified by hospital or non-hospital facility type. We imputed regional averages by facility tier for 3 missing observations (2 districts with no hospitals with a delivery observation, 1 district with no non-hospitals with a delivery observation). We assigned each woman from the MICS with a live birth in the past 2 years an adjusted coverage score based on her reported delivery facility type and district of residence; these scores capture likelihood of receipt of post-delivery intervention based on geographic area and delivery facility type. Women delivering outside of the formal health system were scored as 0.

### Covariates

We extracted household and maternal characteristics from the MICS data, including rural location, household wealth quintile based on an asset index, maternal education (none, primary, secondary and above), maternal age, parity, and birth spacing in months. We calculated the square of maternal age to account for nonlinear associations between maternal age and infant outcomes. Birth interval is set to zero for first births.

### Statistical analysis

To account for missing data due to incomplete MICS responses (aside from birthweight) or lack of spatial coordinates for the EA, we calculated inverse probability weights for each individual-level outcome. We fit logistic regression models with the outcome of complete data and covariates of quality-adjusted coverage, household wealth index, maternal age, primiparity, and birth spacing and predicted the probability each observation was complete. For complete observations, we calculated the inverse probability of being observed to upweight observations most similar to the missing cases. This method relies on the assumption of missing at random: that the missing cases are exchangeable with the observed cases conditional on the observed covariates. We rescaled weights to match the analytic sample size and, for descriptive analysis, multiplied the inverse probability weight with the MICS survey sampling weight. For adjusted analysis, we used only the inverse probability weight.

We present descriptive statistics of the sample of recent singleton births, including proportions for binary and categorical variables and median and IQR for continuous variables; all analyses are weighted to account for missing data and sample design. We describe the frequency of interventions during ANC using the individual direct observations of care, weighted with the SPA survey sampling weight. We map median levels of crude and quality-adjusted coverage during ANC at the district level for illustration.

In adjusted analysis, we fit generalized linear models with a log link to estimate the relative risk of low birthweight associated with first crude coverage and then quality-adjusted coverage of nutrition interventions. Models are adjusted for rural location, household wealth quintile, maternal age, maternal age squared, maternal education, birth spacing, and first child. Models for low birthweight account for the variance introduced by multiple imputation. We fit the same models with the outcome of immediate breastfeeding and test the exposures of crude coverage (number of ANC visits, facility delivery) and quality-adjusted coverage of breastfeeding-related interventions during ANC and intrapartum care. These models are weighted to account for missing data and provide clustered standard errors due to repeated sampling within EAs.

### Ethical approval

The original survey implementers obtained ethical approvals for data collection; the Harvard University Research Protection Program deemed this analysis exempt from human subjects review.

## RESULTS

### Content and quality of nutrition interventions

Of the 1060 facilities on a master list maintained by the Ministry of Health at the time of the SPA survey, 83 were closed, inaccessible, or refused assessment; 977 facilities were successfully assessed (92%), of which 643 provided ANC services and 540 provided normal delivery care. A total of 2068 ANC visits and 474 normal deliveries were directly observed, yielding a median of 5 ANC observations and 1 delivery observation per facility.

**Figure 1:**
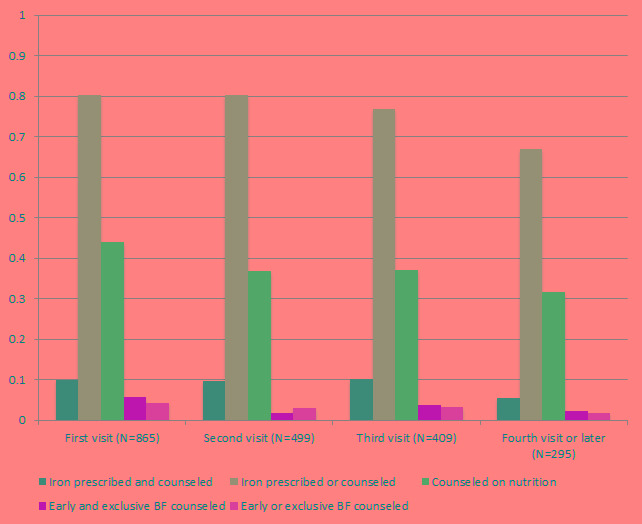
Delivery of nutrition-related interventions during ANC visits (N = 2068 directly observed visits). ANC – antenatal care, BF – breastfeeding.

**Figure 1** shows the delivery of nutrition interventions during directly observed ANC visits. Only 10% of women in the first through third visit and 5% in fourth or later visits had IFA prescribed with appropriate counseling; between 80% (first visit) and 67% (fourth or later visit) of women received an iron prescription alone or counseling without prescription. Counseling on appropriate nutrition during pregnancy was observed in 44% of first visits but only 32% of fourth or later visits. Counseling on early or exclusive breastfeeding was rarely observed: during first visits, only 5.7% of women were counseled on early and on exclusive breastfeeding and 4.2% were counseled on one of these, with even lower frequencies in later visits.

Across the 474 observed deliveries, 90.6% of mothers were seen to initiate breastfeeding within an hour of delivery, 78.9% of newborns were placed directly skin-to-skin, and 91.3% of mother-newborn dyads were kept in the same room following delivery.

### Coverage of maternal care

The MICS sample included 7576 women with a live birth in the past two years, including 173 women delivering twins. We excluded 104 women living in EAs with undefined location, 76 who did not report a specific number of ANC visits, 1 with no information on educational attainment, and 10 cases of multiple births without valid time of breastfeeding initiation. The remaining 7385 observations were eligible for one or both analyses (birthweight and/or breastfeeding). The analysis for which birthweight was the outcome included 7225 singleton live births (97.6% of singleton births in the sample), 6123 (84.7%) of which had birthweight reported based on a health card or maternal recall. The immediate breastfeeding analysis included 7235 live births with valid self-reported time of breastfeeding initiation (95.5% of births in the sample); a single observation was included for each twin delivery as maternal report of breastfeeding was constant within twin sets.

As shown in **Table 1**, the majority of the 7385 women delivering in the past 2 years in the analytic sample lived in rural areas (88%) and had a primary education (71%). Nearly a quarter of recent births were in households in the poorest wealth quintile. As expected, health care utilization was high, with 98% of women seeking any antenatal care (45% at least four visits) and 91% of children delivered in a health facility. Of the 6123 newborns with birthweight reported, 933 (15.5%) weighed 2.5 kg or less. Nearly 80% of newborns were breastfed within one hour per maternal report.

**Table 1:** Characteristics of live births past 2 y (N = 7385)

	N	%
Rural	6510	88.2%
Maternal education:		
None	855	11.6%
Primary	5241	71.0%
Secondary and above	1289	17.5%
Household wealth quintile:		
1 (Poorest)	1824	24.7%
2	1653	22.4%
3	1536	20.8%
4	1230	16.7%
5 (Wealthiest)	1142	15.5%
First child to this mother	1666	22.6%
Child delivered in health facility	6705	90.8%
Mother attended any antenatal care visits	7218	97.7%
Mother attended at least 4 ANC visits	3333	45.1%
Low birth weight (N = 6123)	933	15.5%
Breastfed immediately (N = 7235)	5628	77.8%
	**Median**	**IQR**
Birth spacing (months, if not first child, N = 5534)	40	31-55
Maternal age at birth (years)	25.5	20.8-30.8
Number of times mother attended ANC	3	3-4
Iron-folate interventions in ANC	1.59	1.22-1.99
Nutrition counseling in ANC	1.01	0.57-1.68
Breastfeeding counseling in ANC	0.06	0.00-0.20
Composite birthweight-related interventions in ANC (iron-folate and nutrition)	1.36	1.00-1.79
Composite breastfeeding-related interventions in ANC (breastfeeding counseling and nutrition)	0.57	0.33-0.97
Facility delivery with immediate breastfeeding	1.00	0.79–1.00
Facility delivery with breastfeeding interventions (immediate breastfeeding, rooming in, skin to skin)	0.88	0.67-0.97

ANC – antenatal care, IQR – interquartile range

### Crude and adjusted coverage of ANC

After adjustment for nutrition-related quality of ANC in the service environment, women received nutrition interventions considerably less often than they sought care: over the course of ANC women attended a median of 3 ANC visits but received a median of 1.6 interventions on IFA (either prescriptions for supplements or counseling on IFA adherence and side effects with and without IFA), 1 instance of counseling on diet during pregnancy, and 0.06 instances of counseling on optimal breastfeeding. Women thus received a median of 1.35 maternal nutrition interventions and 0.57 interventions that might increase uptake of breastfeeding.

**Figure 2:**
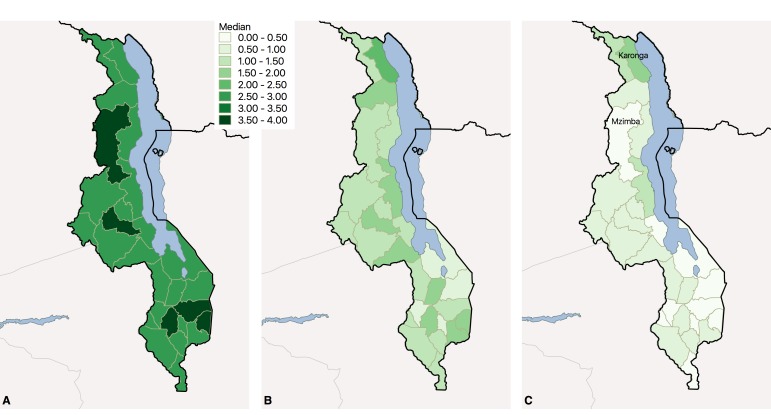
Nutrition interventions during ANC in Malawi, 2013 – 2014. **Panel A.** ANC utilization: Median ANC visits among women with live singleton birth in past 2 years by district. **Panel B.** Nutrition interventions: Median interventions related to maternal nutrition (IFA, nutrition counseling) during ANC visits among women with live singleton birth in past 2 years by district. **Panel C.** Breastfeeding interventions: Median interventions related to breastfeeding (maternal nutrition counseling, breastfeeding counseling) during ANC visits among women with live singleton birth in past 2 years by district. ANC – antenatal care, IFA – iron-folic acid.

**Figure 2** provides a geographic summary of crude ANC coverage and coverage adjusted for receipt of nutrition interventions and breastfeeding interventions at the district level. Women accessed ANC at high levels across the country (**Figure 2**, panel A). Despite this utilization, women were receiving relatively few nutrition interventions that can improve infant birthweight (**Figure 2**, panel B) or improve breastfeeding uptake (**Figure 2**, panel C). Because coverage of nutrition interventions varies, access to ANC does not directly correspond to complete care. For instance, within the Northern Region, Karonga district bordering Lake Malawi had a median of 3 ANC visits per woman (average of 3.4) but provides a median of 2.4 maternal nutrition interventions within those visits and 1.5 breastfeeding interventions, the highest in the country. In contrast, nearby Mzimba district had 4 median ANC visits but only 1.2 maternal nutrition interventions and 0.4 breastfeeding interventions; inferring receipt of nutrition-related interventions from crude ANC coverage can lead to erroneous conclusions. In all districts, the number of ANC visits exceeded the provision of essential nutrition interventions.

### Crude and adjusted coverage of delivery care

**Figure 3:**
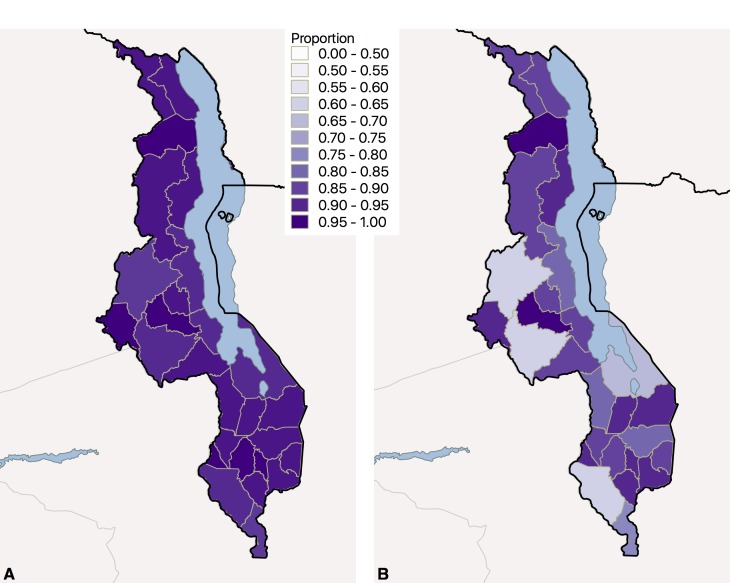
Nutritional interventions at childbirth in Malawi, 2013-2014. **Panel A.** Facility delivery. **Panel B.** Facility delivery with immediate breastfeeding.

**Figure 3** provides a geographic summary of delivery coverage and coverage adjusted for immediate breastfeeding at the district level, again showing high levels of health service utilization in panel A. While a few districts show droped between facility delivery and delivery with observed immediate breastfeeding (**Figure 3**, panel B), many districts achieved high coverage and high adjusted coverage.

### Association of coverage with health outcomes

Both crude and quality-adjusted ANC coverage were associated with lower risk of low birthweight newborns in multivariable regression (**Table 2**, panel A). The risk of a low birthweight newborn is 10% lower with each additional ANC visit and 13% lower with each additional nutrition-related intervention (adjusted relative risk (ARR) = 0.87, 95% confidence interval (CI) = 0.79, 0.96). These models suggest that if women received all of the recommended nutrition interventions at each ANC visit they currently attend, the population prevalence of low birthweight would decrease by almost 3 percentage points, from 13.7% (95% CI = 13.6, 13.8) to an estimated 10.8% (95% CI = 10.7, 10.9).

**Table 2:** Association of coverage and quality-adjusted coverage with newborn health outcomes

	Relative risk	95% confidence interval
**A: Low birthweight (N = 7225):**		
Model A1:*		
Number of ANC† visits	0.90	0.85, 0.95
Model A2:*		
Number of nutrition-related interventions in ANC	0.87	0.79, 0.96
**B: Immediate breastfeeding (N = 7235):**		
Model B1:*		
Number of ANC visits	1.01	1.00, 1.02
Facility delivery	1.06	1.00, 1.13
Model B2:*		
Number of breastfeeding-related interventions in ANC	1.04	1.02, 1.07
Facility delivery with immediate breastfeeding	1.08	1.02, 1.14

ANC – antenatal care

*All models are controlled for: rural/urban location, maternal age at birth and age squared, wealth quintile, maternal education (none, primary, secondary or greater), first birth, birth spacing (months). Models are weighted to account for observations excluded due to missing data; confidence intervals account for clustering due to repeated samples within enumeration area. Models with low birthweight as an outcome are based on 5 data sets with multiple imputation for missing birthweight.

Crude and adjusted coverage of ANC and delivery are also associated with greater likelihood of immediate breastfeeding in models adjusted for demographic and individual characteristics (**Table 2**, panel B). Nutrition quality-adjusted measures show slightly stronger links: the likelihood of initiating breastfeeding immediately is 4% higher with each additional breastfeeding intervention received during ANC (ARR = 1.04, 95% CI = 1.02, 1.07) and 8% higher for facility delivery with breastfeeding interventions compared to women without quality care at delivery (ARR = 1.08, 95% CI = 1.02, 1.14). This suggests that the population prevalence of immediate breastfeeding would increase from 75.9% (95% CI = 75.8, 76.0) to 86.0% (95% CI = 85.8, 86.1) if women received nutrition-related interventions in their current ANC visits and facility delivery; almost all of this potential increase (9.7 percentage points) is attributed to increased quality of ANC given the already high prevalence of immediate breastfeeding for women delivering in health facilities. Results are essentially unchanged when using three indicators (observation of immediate breastfeeding, rooming in, and placing newborn skin to skin) to adjust coverage for post-delivery care (not shown).

## DISCUSSION

In low and middle-income countries, one in four neonatal deaths occur in low birthweight infants [40,41]. Maternal nutrition supplementation and counseling during ANC and support for immediate initiation of breastfeeding after delivery are important interventions for improving birthweight and reducing neonatal mortality. Household surveys do not consistently and reliably capture data on coverage of these interventions [34,42], and reported crude coverage estimates, such as number of ANC visits or facility delivery, do not capture the content of these services or their quality.

In this study, we applied a method for incorporating nutrition interventions into coverage estimates of ANC and post-delivery care by linking observed data from health facility service provision assessments to self-reported health care utilization from household surveys. These individual-level adjusted coverage metrics were associated with lower risk of low birthweight newborns and higher probability of immediate breastfeeding, demonstrating their salience to population health outcomes. An additional ANC visit including nutritional interventions was associated with a 13% lower risk of low birthweight compared to a 10% reduction for an additional ANC visit without considering nutrition interventions. Based on these models, if women were to receive all recommended nutrition interventions during antenatal care, population prevalence of low birth weight would decrease by an estimated 3 percentage points, from 14% to 11%. If effected, this 21% relative reduction in low birth weight could reduce neonatal mortality at a population level.

Nutrition-adjusted coverage interventions for both ANC and post-delivery care were also associated with greater likelihood that mothers will initiate breastfeeding within 1 hour of birth. The likelihood of initiating immediate breastfeeding was 4% higher with each additional breastfeeding intervention received during ANC and 8% higher for delivery in a facility with breastfeeding interventions. Ensuring breastfeeding-related interventions are delivered as intended within existing ANC and delivery care could lead to an estimated 10% increase (76% to 86%) in the prevalence of immediately breastfed infants, largely driven by potential improvements in the content of ANC. Immediate breastfeeding is associated with substantial mortality reductions in all infants and among those exclusively breastfed [7]; an increase in population rates of immediate breastfeeding can contribute to newborn survival [43].

Findings from this study suggest substantial population health benefits from a focus on health care quality given the high levels of utilization already achieved. This expands on prior work evaluating access and quality of care in Malawi [31,44] and underscores the government’s emphasis on quality improvement as a health sector priority, particularly within the Maternal-Neonatal Health section [45-47]. While the gap between utilization and quality (provision of evidence-based interventions) is notable in Malawi, other studies have documented deficits in care content for ANC even when utilization is high [29,34,48-51]. Self-reported 90-day coverage of iron for pregnant women was 33% in 2015-2016 [52]; while women may obtain iron outside the formal health sector or without prompting from a clinician, the deficit even in self-reported coverage supports our finding of missed opportunities to improve health. Scalable strategies to increase intervention delivery are needed to address these gaps. For example, while stock outs of iron folate could shape provider behaviors on prescribing and counseling, breastfeeding promotion, which was done rarely by health providers in this study, does not require intensive resources, can be delivered at all levels of the health system and can be performed by competent health workers or in the group setting at any ANC visit [53].

Quality-adjusted and effective coverage metrics for maternal, newborn, and child health and nutrition are necessary to benchmark and guide the type of health system strengthening that can result in better population health by addressing both access and quality [18,19,54]. Development of valid and reliable metrics has been limited to date due to the reliance on household surveys. Self-reported measures of content of care may be unreliable when recall pertains to services or advice rendered during labor and delivery, when recall periods are prolonged, or when care pertains to socially desirable behaviors [35,36,42]. This study is the first to develop and apply nutrition-related metrics of effective coverage for antenatal and labor care and to demonstrate the relevance of such metrics for population health outcomes. It employs a novel methodology of calculating quality-adjusted coverage at the individual and population level by combining health facility and population assessments using exact geographic information and employing direct observation of care to provide reliable estimates of delivery of nutrition interventions. The results underscore the importance of delivering these interventions with quality to improve maternal and neonatal outcomes.

From a measurement perspective, this study was possible due to the contemporaneous SPA and MICS surveys in Malawi and the availability of geographic information to link them. Relatively few instances of overlapping surveys exist to date. Data from health facility assessments such as the SPA are under-utilized, and there are far fewer of these surveys than DHS and MICS. To make the best use of these data sources, there is a need to coordinate the data collected [55], both at a broad scale of survey timing and locations as well as at a granular level in collecting more details on household surveys regarding type of facility used and whether it is the closest facility of that type to enable closer linking. Efforts to align health facility surveys such as the SPA, Service Delivery Indicators, and Service Availability and Readiness Assessment to provide a common basis for content of care assessment are an important step [56]. Ongoing monitoring of effective coverage will demand fresh thinking around incorporating routine data sources, such as linking detailed quality information like the SPA to sparser but more frequently measured elements such as health management information systems (HMIS). Further research on methods to enhance and make use of routine data collection is warranted.

There are several important limitations to our study. One of the primary outcomes was birthweight: we imputed birthweight for 15% of the observations due to missing data, relying on the assumptions that the data were missing at random and that the imputation model was correctly specified. A novel aspect of this work is our approach to linked health service provision data and household survey information in order to come up with quality adjusted metrics for individual women. Our data sources were not exactly contemporaneous – the MICS was carried out in 2013-2014 and the SPA was done in 2013. Due to the recall period for live birth, ANC visits may have taken place up to 2 years and 9 months before the survey; recall may be imperfect and quality of care may vary across this time period. We were not able to explicitly link mothers to specific facilities based on available data and relied on average quality across the nearby service environment; estimates of delivery care quality were based on observations of deliveries during the facility assessment, meaning less information is available on low-volume facilities than high-volume facilities. Finally, given the observational data, residual confounding due to unmeasured variables may bias the results.

Our findings show it is feasible to combine health facility and population data to incorporate quality into crude coverage measures in order to address the coverage and quality of nutrition interventions as part of antenatal, delivery, and newborn care. The findings are in line with results from clinical studies that demonstrate health impact of nutrition interventions on breastfeeding practices and low birth weight [53,57]. The results show that quality-adjusted measures demonstrate clear links to individual health outcomes of global health importance, supporting their utility for health system assessment. These findings also suggest that improvements in quality may have the potential to reduce neonatal mortality in Malawi within the existing high levels of health system coverage.
